# Prediction of Protein–Protein Interactions in *Arabidopsis*, Maize, and Rice by Combining Deep Neural Network With Discrete Hilbert Transform

**DOI:** 10.3389/fgene.2021.745228

**Published:** 2021-09-20

**Authors:** Jie Pan, Li-Ping Li, Zhu-Hong You, Chang-Qing Yu, Zhong-Hao Ren, Yong-Jian Guan

**Affiliations:** School of Information Engineering, Xijing University, Xi’an, China

**Keywords:** deep neural networks, discrete hilbert transform, plant, protein–protein interactions, position-specific scoring matrix

## Abstract

Protein–protein interactions (PPIs) in plants play an essential role in the regulation of biological processes. However, traditional experimental methods are expensive, time-consuming, and need sophisticated technical equipment. These drawbacks motivated the development of novel computational approaches to predict PPIs in plants. In this article, a new deep learning framework, which combined the discrete Hilbert transform (DHT) with deep neural networks (DNN), was presented to predict PPIs in plants. To be more specific, plant protein sequences were first transformed as a position-specific scoring matrix (PSSM). Then, DHT was employed to capture features from the PSSM. To improve the prediction accuracy, we used the singular value decomposition algorithm to decrease noise and reduce the dimensions of the feature descriptors. Finally, these feature vectors were fed into DNN for training and predicting. When performing our method on three plant PPI datasets *Arabidopsis thaliana*, maize, and rice, we achieved good predictive performance with average area under receiver operating characteristic curve values of 0.8369, 0.9466, and 0.9440, respectively. To fully verify the predictive ability of our method, we compared it with different feature descriptors and machine learning classifiers. Moreover, to further demonstrate the generality of our approach, we also test it on the yeast and human PPI dataset. Experimental results anticipated that our method is an efficient and promising computational model for predicting potential plant–protein interacted pairs.

## Introduction

Identification of protein–protein interactions (PPIs) in plants is essential for exploring the mechanisms underlying of biological processes, such as organ formation, homeostasis control (Canovas et al., 2004), plant defense (Zhang et al., 2010), signal transduction (Khan and Kihara, 2016), and stress response (Bracha-Drori et al., 2004). Although numerous high-throughput techniques have been developed to identify PPIs of model species, such as affinity purification mass spectrometry (Fukao, 2012; Armean et al., 2013) and yeast two-hybrid (Causier and Davies, 2002; Fang et al., 2002), these approaches are cumbersome, costly, particularly time consuming, and always suffer from high false positive rate. To overcome these problems, there is an urgent need to develop sequence-based computational methods that can accurately predict potential PPIs while analyzing the functions of plant genes.

In recent years, many studies have been introduced for detecting PPIs. These methods can be broadly classified into several categories: protein structure–based method (Hayashi et al., 2018), genomic information–based method (Zahiri et al., 2014), evolutionary relationship–based approach (Xu et al., 2011), and protein sequence–based method (Richoux et al., 2019). In fact, the first three methods have better prediction performance. However, these methods typically require the structural details of proteins such as 3D structural and protein homology information. If this prior knowledge is not available, then the method will not perform as expected. Theoretically, amino acid sequence contains all the necessary information to detect PPIs. In addition, with the improvement of sequencing technology, more and more plant genome sequences are available. Hence, it is meaningful to develop computational methods to predict potential PPIs from sequence information.

To date, some new approaches have been proposed to predict PPIs using the feature descriptors of protein sequence, such as the composition-transition-distribution descriptor (Yang et al., 2010), auto-covariance descriptor (Guo et al., 2008), Zernike moments descriptor (Wang et al., 2017), and local descriptor (Davies et al., 2008). These descriptors summarize specific aspects of amino acid sequence, including frequencies of local patterns, physicochemical properties, and positional distribution of protein sequence. However, the coverage of these feature descriptors is still limited. Recently, many deep learning techniques also have been applied on PPI-based prediction. For example, Du et al. (2017) presented an approach called DeepPPI, which adopted deep neural networks (DNN) to extract high-level features from raw input features of protein sequence to identify PPIs. Zeng et al. (2020) were inspired by the deep learning algorithm and proposed a framework called DeepPPISP, which extracts local and global features from amino acid sequences and employs DNN to predict PPIs. Sun et al. (2017) employed stacked autoencoder (SAE), which is a deep learning algorithm to predict PPIs from human protein sequence. Hashemifar et al. (2018) developed a novel sequence-based approach called DPPI that used Siamese-like convolutional neural networks (CNN) combined with data augmentation and random projection to improve PPI prediction. Sledzieski et al. (2021) proposed a novel model named D-SCRIPT, which indicated that employing a deep learning language modeling of protein sequence data is effective for PPI prediction. Chen et al. (2019) put forward an end-to-end framework that combined contextualized information and local features with a deep residual recurrent CNN in the Siamese architecture to predict PPIs only using protein sequence information. Yi et al. (2018) proposed the RPI-SAN model using a deep learning stacked autoencoder network to extract features from RNA and amino acid sequences. Finally, they fed these features to the RF model for training and predicting. Despite these advances in previous studies, there is still a need to improve the accuracy and efficiency of the PPI prediction models.

In this article, we combined DNN with discrete Hilbert transform (DHT) and singular value decomposition (SVD) to predict PPIs in plants. More specifically, for each plant primary sequence, position-specific score matrix (PSSM) was constructed, and then DHT was applied to gather important information from the protein PSSM. Subsequently, SVD algorithm was adopted to reduce feature dimension and noise interference and finally generated a 600-dimensional feature vector. Lastly, a deep neural network was applied to make predictions between target plant proteins. When the proposed method was applied on the *Arabidopsis thaliana*, maize (*Zea mays*), and rice (*Oryza sativa*) PPI datasets, it yielded promising results of average AUC (area under ROC curve) values of 0.8369, 0.9466, and 0.9440. When compared with some different feature selection methods and state-of-the-art machine learning classifiers, our method obtained better results. In addition, to achieve more convincing evidence, we also applied our method to the yeast and human PPI dataset. These combined results suggest that the proposed approach is effective and trustworthy for predicting potential PPIs in plants.

## Materials and Methods

### Data Collection and Construction of the Benchmarking Set

To validate the robustness and effectiveness of the proposed model, we performed it on three plant PPI datasets, *A. thaliana*, *Z. mays*, and *O. sativa*. The *A. thaliana* dataset was collected from TAIR^1^ (Rhee et al., 2003), IntAct^2^ (Kerrien et al., 2012), and BioGRID^3^ (Stark et al., 2006). After removing the redundancy, the final *A. thaliana*–positive dataset comprised 28,110 PPI pairs containing 7,437 *A. thaliana* proteins. These protein-interacted pairs constructed the primary *A. thaliana* PPI network. For the construction of the negative dataset, we employed a bipartite to formulate a network of plant PPIs, where the nodes represent the plant proteins and the links denote the interactions between them. Here, we use *A. thaliana* as an example. The whole associations between the 7,437 proteins are 55,308,969 (7,437 × 7,437) in the corresponding bipartite. However, only 28,110 PPIs had been demonstrated to have the interactions. Thus, the possible number of negative pairs is 55,280,859 (55,308,969–28,110), which is significantly more than the positive samples. To handle this binary classification problem, we randomly collected 28,110 non-interacting pairs as the negative dataset. In theoretical terms, the negative samples may contain a small number of positive samples; however, given the size of the whole non-interaction pairs, the probability of this situation is very small. In this way, the whole *A. thaliana* dataset consists of 56,220 protein pairs.

Maize and rice are the main cash crops in the world. The maize (*Z. mays*) dataset contains 14,800 positive pairs, which was downloaded from PPIM^4^ (Zhu et al., 2016) and agriGO^5^ (Tian et al., 2017). Similarly, we assumed that the proteins in different subcellular work compartments have no interactions and finally achieved 14,800 non-interacting protein pairs. The rice (*O. sativa*) dataset consisted of 9,600 protein pairs, 4,800 positive pairs, and 4,800 negative pairs collected from the PRIN database^6^ (Gu et al., 2011).

### Representation of the Plant Amino Acid Sequence

To mine highly efficient features for training the models, each protein pair is encoded as 800-dimensional feature vector by PSSM (Gribskov et al., 1987). PSSM has been successfully employed in various fields of biological research including the prediction of PPI site, subcellular localization, and DNA-binding protein identification. In this section, we applied PSI-BLAST (Altschul and Koonin, 1998) tool to represent protein sequence as a *U* × 20 matrix, where *Q* = {*η*_*a*,*b*_:*a* = 1⋯*U* *and*b = 1⋯20}, and it can obtain the information of plant sequential evolution. PSSM can be defined as


(1) $$Q=\left[\begin{array}{c} \eta_{1,1},\eta_{1,2},\cdots \,\eta_{1,20}\\ \eta_{2,1},\eta_{2,2},\cdots \,\eta_{2,20}\\ \vdots \;\vdots \;\cdots \;\cdots \\ \eta_{U,1},\,\,\,\eta_{1,2},\cdots \,\eta_{U,20} \end{array}\right]$$

where η_*a,b*_ represents probability that the *a-*th mutate to *b-*th amino acid during the evolutionary process. In the experiment, plant protein sequences were adopted as seeds to search and align homogenous sequences from SwissProt database by PSI-BLAST tool. The tool will be used to recognize members of gene family and evolutionary relationships between plant protein sequences. It is also able to generate a 20-dimensional vector to denote the probabilities of conservation against mutations to the 20 amino acids. The number of iterations is set to 3 and the *E*-value is cut off at 0.001 to achieve homologous sequences. The PSI-BLAST tool and SwissProt database can be accessed online^7^.

### Discrete Hilbert Transform

In this section, we introduce discrete Hilbert transform (DHT; Cizek, 1970) to extract feature descriptors from the PSSM to make the prediction more convenient and accurate. DHT is used as a tool for signal analysis in the time and frequency domains. Before describing the 2-dimensional DHT, the 1-D DHT (Ponomareva et al., 2018) is used in the spatial and frequency domain and has been previously described (Stark, 1971; Bracewell and Bracewell, 1986; Zhu et al., 1990; Onodera et al., 2005).

To better extract the feature descriptors, we used the 2-D DHT for constructing the local energy of PSSM. In this work, we applied the 2-D DHT, which is defined by Read and Treitel (1973) in the frequency domain. Our Matlab code is shown as follows:

function x = hilbert2(xr,m,n)

%HILBERT2 Discrete-time 2D analytic signal via Hilbert transform.

% X = HILBERT2(Xr) computes the 2D discrete-time analytic signal

% X = Xr + i^∗^Xi such that Xi is the Hilbert transform of real image Xr.

% If the input Xr is complex, then only the real part is used: Xr = real(Xr).

% HILBERT2(Xr,M,N) computes the MxN-point Hilbert transform. Xr is padded

% zeros if it has less than MxN points, and truncated if it has more.

if nargin < 2, n = []; end

if ∼isreal (xr)

   warning (’HILBERT2 ignores imaginary part of input.’)

   xr = real (xr);

end

if isempty (n)

   [m, n] = size (xr);

end

if *m* < 2 | | *n* < 2,

   x = Hilbert (xr); % 1D analytic signal

   return;

end;

In this work, PSI-BLAST encoded each protein sequence as a *U*×20 matrix. Due to the different lengths of protein sequences, the size of each matrix constructed by PSSM is also different. To handle this problem, we transformed the variably sized PSSM into a 20×20 matrix, and the 2-D DHT is applied to extract feature vectors from the PSSM profile. In this way, each plant protein sequence will be converted into a 400-dimensional vector by 2-D DHT. As a non-linear filtering technique, SVD has been widely applied in noise reduction of vibration signals. This is because the signals after noise reduction have a small phase-shift and there is no time delay effect. To improve the prediction accuracy and reduce the dimensionality of the input feature matrix, we applied SVD (Klema and Laub, 1980) algorithm to reduce size of feature vectors from 400 to 300. At the same time, the lower dimensions could reduce the complexity of the model and increase the generalization error of the classifier. Finally, each protein pair will be represented as a 600-dimensional DHT descriptor.

### Deep Neural Networks

**FIGURE 1:**
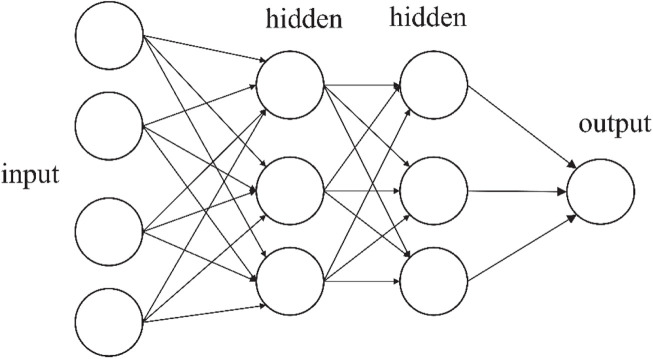
The construction of deep neural networks.

Considering the larger numbers of hidden layers that can be used for training networks, artificial neural networks consist of two or more hidden layers that are often referred as DNN as shown in Figure 1. The depth of a neural network relates to the quantity of hidden layers, and the largest number of neurons determines the width of DNN (Hinton et al., 2006; Hinton and Salakhutdinov, 2006).

In terms of structure, DNN is composed of many plain modules, which appear as a multilayer stack. The data are first received by the input layer, and then converted through a non-linear way across many hidden layers. Before calculating the final output, the average gradient is first computed and the corresponding weights are adjusted. Neurons of a hidden layer or input layer are associated with the neurons of the existing layer. Each neuron will compute a weighted sum of its input and perform a non-linear activation function to capture its outputs. The non-linear activation functions usually include sigmoid, rectified linear unit (ReLU), and hyperbolic tangent. In this work, we used the sigmoid and ReLU. We constructed a DNN-based model using the TensorFlow platform shown in Figure 1. This model consists of two hidden layers with 48 neurons each. The DHT feature descriptors are employed as the inputs for the DNN model. After that, these features were set into the hidden layers for training and predicting PPIs. Adam algorithm (Kingma and Ba, 2014), which is an adaptive learning rate approach, was adopted in our methods to accelerate the training process. At the same time, to avoid overfitting, the dropout technique was also applied to our model (Khan et al., 2019). We also used the cross-entropy loss and ReLU activation function to speed our training and achieve better predictive performance (Hinton et al., 2015). The loss can be calculated by the following formulas:


(2) $$R_{i\,1}^{m}=\sigma \left(T_{i\,1}X_{i\,1}+b_{i\,1}\right)\left(i=1,2,3,\cdots ,n;m=1,2\right)$$

$$R_{i\,j}^{m}=\sigma \left(T_{i\,j}R_{i\,\left(j-1\right)}+b_{i\,j}\right)(i=1,2,3,\cdots ,n;$$

(3) $$j=2,3,4\cdots ,h_{1};m=1,2)$$

(4) $$R_{i\,k}^{3}=\sigma_{1}\left(T_{i\,k}\left(R_{i\,h_{1}}^{1}⊕R_{i\,h_{1}}^{2}\right)+b_{i\,k}\right)\left(i=1,\cdots ,n;k=h_{1}+1\right)$$

(5) Ri⁢k3=σ1(TRi⁢(k-1)i⁢k+bi⁢k)(i=1,⋯,n;k=h1+2,⋯,h2)

$$L=-\frac{1}{n}\sum_{i=1}^{n}[y_{i}\ln(\sigma_{2}\left(T_{i\,h\,2}R_{i\,h\,2}+b_{i\,h\,2}\right)$$

(6) $$+\left(1-y_{i}\right)\ln\left(1-\sigma_{2}\left(T_{i\,h\,2}R_{i\,h\,2}+b_{i\,h\,2}\right)\right)]$$

In Eqs. 2–6, *n* describes the amount of protein pairs that need to be trained, *m* denotes the individual network, *h*_*1*_ represents the depth of two individual networks, and *h*_*2*_ denotes the depth of the fused network. The activation function of ReLU and output layer with sigmoid is σ_*1*_ and σ_*2*_, respectively; ⊕ is the concatenation operator. *R* represents the output of hidden layer and *y* is the corresponding desired output. *T* and *b* indicate the weight matrix and bias vectors.

## Results

### Evaluation Criteria

To prevent overfitting and validate the robustness of our method, five-fold cross-validation (CV) scheme is performed on our method. Specifically, the entire plant’s PPI dataset will be randomly split into five equal parts; four of them will be employed for training and the remaining one was used for testing. The training and testing data will not overlap with each other to prevent overfitting. The final validation results were the mean value obtained by the five-fold CV scheme. The predictive performance of the proposed approach is verified by five different measurements, including accuracy (Acc), precision (PR), sensitivity (Sens), specificity (Spec), and MCC. They can be represented by


(7) $$A\,c\,c=\frac{T\,P+T\,N}{T\,P+F\,P+T\,N+F\,N}$$

(8) $$P\,R=\frac{T\,P}{T\,P+F\,P}$$

(9) $$S\,e\,n\,s=\frac{T\,P}{T\,P+F\,N}$$

(10) $$S\,p\,e\,c=\frac{T\,N}{F\,P+T\,N}$$

(11) $$M\,C\,C=\frac{T\,N\times T\,P-F\,P\times F\,N}{\sqrt{\left(T\,P+F\,P\right)\times \left(T\,P+F\,N\right)\times \left(T\,N+F\,N\right)\times \left(T\,N+F\,P\right)}}$$

where TP, FP, TN, and FN are associated with the number of true positive, false negative, true negative, and false negative, respectively. In addition, receiver operating characteristic (ROC) curves (Hand, 2009) were plotted for better accessing the predictive performance of the proposed model. Furthermore, AUC (area under ROC curve) Huang and Ling (2005) values were also used as an evaluation criterion.

### Predictive Performance of Our Model on Three Plant Datasets

**FIGURE 2:**
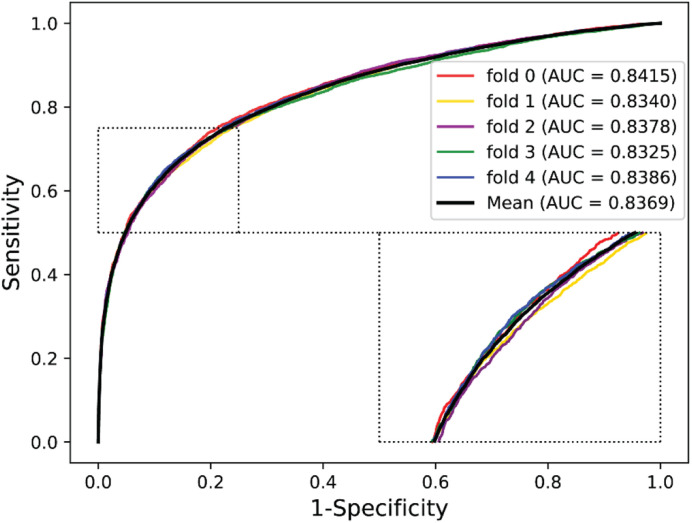
The ROC curves of our approach on the *A. thaliana* dataset under five-fold CV.

**FIGURE 3:**
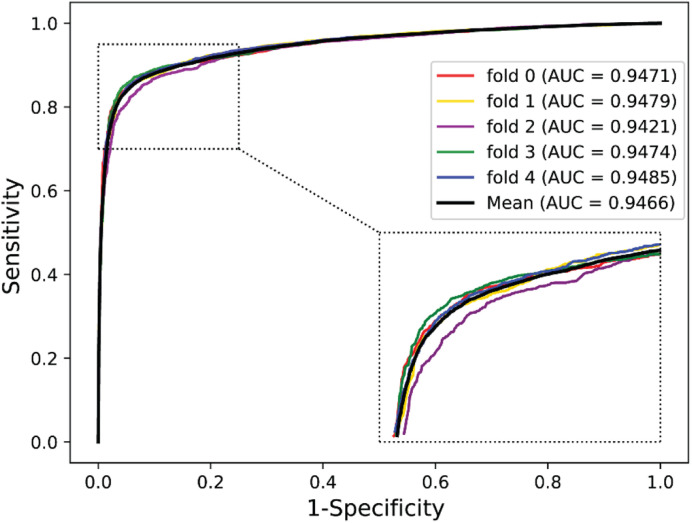
The ROC curves of our approach on the *Zea mays* dataset under five-fold CV.

**FIGURE 4:**
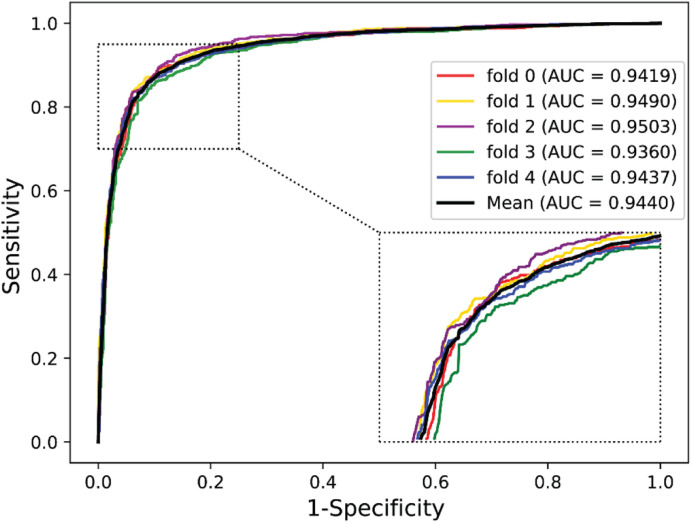
The ROC curves of our approach on the *Oryza sativa* dataset under five-fold CV.

We validated the predictive performance of the proposed model on three plant PPI datasets by five-fold CV scheme, including *A. thaliana*, *Z. mays*, and *O. sativa*. It can be observed from Table 1 that the average accuracy (Acc), precision (PR), sensitivity (Sens), specificity (Spec), and Matthews correlation coefficient (MCC) and AUC values obtained on the *A. thaliana* dataset are 71.48%, 66.64%, 86.09%, 56.88%, 44.94%, and 0.8369, respectively. Their SDs are 0.69, 0.89, 1.08, 2.21, 1.14, and 0.36%, respectively. Table 2 lists the prediction results obtained on the *Z. mays* dataset, from which we can see the average Acc of 85.41%, PR of 81.54%, Sens of 91.67%, Spec of 79.17%, MCC of 71.43%, and AUC of 0.9466, respectively. Their SDs are 1.18, 2.38, 1.18, 3.24, 2.00, and 0.26%, respectively. On the *O. sativa* dataset, shown in Table 3, our model performs at an Acc of 82.60%, PR of 75.79%, Sens of 95.89%, Spec of 69.31%, MCC of 67.65%, and AUC of 0.9442, with SDs of 1.79, 2.43, 0.91, 3.53, 2.98, and 0.58%, respectively. Figures 2–4 illustrate the ROC curves yielded on *A. thaliana*, *Z. mays*, and *O. sativa* datasets. In the figure of ROC curves, *x*-axis is the false positive rate and *y*-axis represents the true positive rate.

**TABLE 1:** Five-fold CV results performed on the *A. thaliana* dataset by the proposed model.

Testing set	Acc (%)	PR (%)	Sens (%)	Spec (%)	MCC (%)	AUC
1	71.54	66.45	87.08	55.98	45.31	0.8415
2	72.05	67.73	84.64	59.36	45.49	0.8340
3	72.25	67.30	85.69	59.03	46.35	0.8378
4	70.87	66.28	85.80	55.73	43.59	0.8325
5	70.71	65.46	87.25	54.30	43.98	0.8386
**Average**	**71.48 ± 0.69**	**66.64 ± 0.89**	**86.09 ± 1.08**	**56.88 ± 2.21**	**44.94 ± 1.14**	**0.8369 ± 0.0036**

**TABLE 2:** Five-fold CV results performed on the *Zea mays* dataset by the proposed model.

Testing set	Acc (%)	PR (%)	Sens (%)	Spec (%)	MCC (%)	AUC
1	84.63	80.07	91.80	77.59	70.04	0.9471
2	84.36	78.90	93.40	75.50	69.95	0.9479
3	85.84	83.41	90.28	81.19	71.87	0.9421
4	84.94	80.73	91.95	77.89	70.56	0.9474
5	87.26	84.59	90.91	83.67	74.74	0.9485
**Average**	**85.41 ± 1.18**	**81.54 ± 2.38**	**91.67 ± 1.18**	**79.17 ± 3.24**	**71.43 ± 2.00**	**0.9466 ± 0.0026**

**TABLE 3:** Five-fold CV results performed on the *Oryza sativa* dataset by the proposed model.

Testing set	Acc (%)	PR (%)	Sens (%)	Spec (%)	MCC (%)	AUC
1	80.21	72.29	96.03	65.28	64.03	0.9419
2	82.60	75.00	96.24	69.74	68.04	0.9490
3	85.05	78.77	96.73	72.93	71.95	0.9503
4	83.33	77.17	94.33	72.49	68.40	0.9360
5	81.82	75.71	96.12	66.12	65.84	0.9437
**Average**	**82.60 ± 1.79**	**75.79 ± 2.43**	**95.89 ± 0.91**	**69.31 ± 3.53**	**67.65 ± 2.98**	**0.9440 ± 0.0058**

Based on the experimental results, it can be indicated that the proposed model is effective for identifying PPIs in plants. We attributed this better prediction performance to the powerful DHT–SVD descriptors and the excellent DNN classifier. The PSSM not only encodes the sequence into matrix but also obtains the sufficient prior information of plant proteins. In addition, the application of DHT extracted robust feature descriptors from PSSM, and then, SVD algorithm was employed to reduce the noise and decrease the dimension of feature matrix that can better improve the prediction performance. As a popular deep learning classifier, DNN shows the powerful ability for training and predicting, which makes us more convinced that our method can be a useful tool for plant PPI prediction.

### Comparison With Random Forest and K-Nearest Neighbor Classifier

**FIGURE 5:**
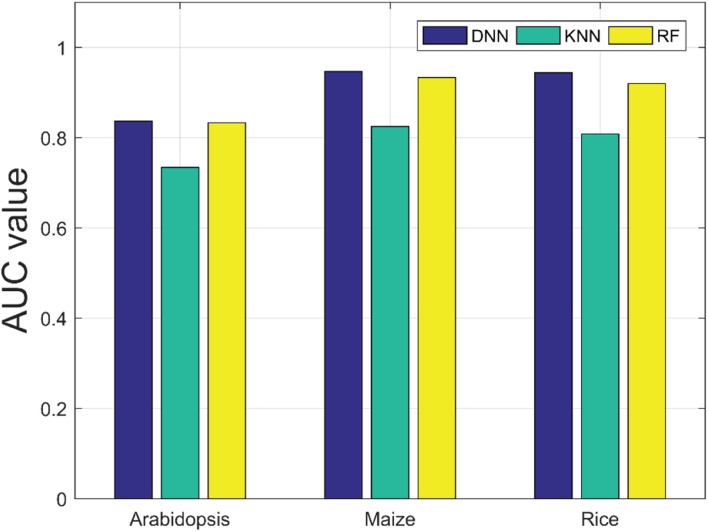
Comparison results of AUC values obtained by deep neural network (DNN), K-nearest neighbor (KNN), and random forest (RF) classifiers on the three plant PPI datasets.

There are many machine learning classifiers that have been applied to predict PPIs. K-nearest neighbor (KNN) (Keller et al., 1985) and random forest (RF) (Breiman, 2001) are the most widely used algorithms. The KNN algorithm is one of the simplest classification approaches and it has been widely applied to detect PPIs (Li et al., 2009). RF is a decision tree–based ensemble learning method, and it is known for its powerful ability of classification (Li et al., 2012). To further verify the predictive ability of DNN classifier, we compared it with the KNN and RF model by the five-fold CV scheme and adopted the same DHT feature descriptors. The results list in Table 4 illustrates that our method achieved higher AUC values across the *A. thaliana*, *Z. mays*, and *O. sativa* datasets. It can be observed that the average AUC values of the DNN classifier are 0.1023, 0.1215, and 0.1354 higher than those of KNN classifier. Similarly, when compared with the RF classifier, the AUC value of our model improved 0.0036, 0.013, and 0.0241, respectively. From the comparison results shown in Figure 5, we considered that the combination of DNN classifier and DHT descriptors can significantly improve the performance in plant PPI prediction.

**TABLE 4:** Five-fold CV results yielded by KNN and RF classifier on the three plant PPI datasets.

Dataset	Classifier	AUC	PR (%)	Sens (%)	Spec (%)	MCC (%)
*A. thaliana*	KNN	0.7346 ± 0.22	71.12 ± 0.44	79.00 ± 0.54	67.92 ± 0.43	60.77 ± 0.22
	RF	0.8333 ± 0.77	82.63 ± 0.94	68.31 ± 1.23	85.63 ± 0.88	64.01 ± 0.69
	Our method	0.8369 ± 0.36	66.64 ± 0.89	86.09 ± 1.08	56.88 ± 2.21	44.94 ± 1.14
*Zea mays*	KNN	0.8251 ± 0.42	78.38 ± 0.77	89.77 ± 0.48	75.25 ± 0.77	70.83 ± 0.57
	RF	0.9336 ± 0.40	96.98 ± 0.28	89.52 ± 0.48	97.21 ± 0.34	87.57 ± 0.49
	Our method	0.9466 ± 0.26	81.54 ± 2.38	91.67 ± 1.18	79.17 ± 3.24	71.43 ± 2.00
*Oryza sativa*	KNN	0.8086 ± 0.89	76.41 ± 1.55	89.28 ± 0.78	72.44 ± 1.58	68.59 ± 1.17
	RF	0.9199 ± 0.58	87.30 ± 1.35	88.00 ± 1.34	87.22 ± 1.16	78.26 ± 1.28
	Our method	0.9440 ± 0.58	75.79 ± 2.43	95.89 ± 0.91	69.31 ± 3.53	67.65 ± 2.98

### Comparison of Position-Specific Scoring Matrix With Different Protein Representation Methods

**FIGURE 6:**
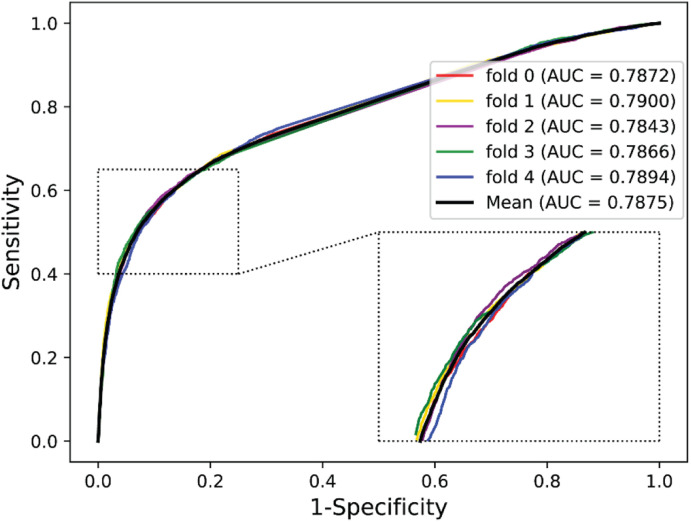
ROC curves obtained from SMR-based method on the *A. thaliana* dataset.

To evaluate the performance of PSSM, we compared it with the substitution matrix representation (SMR), which was proposed by Yu et al. (2012) to represent protein sequence. In this section, we employed the BLOSUM62 matrix to encode the *A. thaliana* protein sequence as a 20 × 20 matrix. Then, the DHT algorithm was applied to extract feature descriptors from SMR matrix and SVD was also adopted to reduce the feature dimensions. By this way, we can generate a 600-dimensional SMR–DHT descriptor for each protein pair. The five-fold CV results of SMR–DHT descriptors combined with DNN classifier on the *A. thaliana* dataset are summarized in Table 5. It can be observed that the PSSM-based method performs significantly better than the SMR-based method. For example, the accuracy and AUC gaps between PSSM and SMR-based method are 4.38 and 4.94%, respectively. The higher predictive accuracy and lower SDs further indicated that our method performs better than the SMR-based approach (Figure 6).

**TABLE 5:** Comparison of PSSM with SMR-based method on the *A. thaliana* dataset.

Testing set	Acc (%)	PR (%)	Sens (%)	Spec (%)	MCC (%)	AUC
1	71.54	71.26	72.27	70.81	43.08	78.72
2	61.05	57.03	90.82	31.04	27.29	79.00
3	58.44	54.79	92.68	24.74	23.70	78.43
4	72.47	74.73	68.51	76.50	45.14	78.66
5	72.02	71.34	73.25	70.80	44.06	78.94
**Average**	**67.10 ± 6.79**	**65.83 ± 9.2**	**79.51 ± 11.34**	**54.78 ± 24.76**	**36.65 ± 10.29**	**0.7875 ± 0.0023**
**Our method**	**71.48 ± 0.69**	**66.64 ± 0.89**	**86.09 ± 1.08**	**56.88 ± 2.21**	**44.94 ± 1.14**	**0.8369 ± 0.0036**

### Comparison With Different Feature Extraction Methods

**FIGURE 7:**
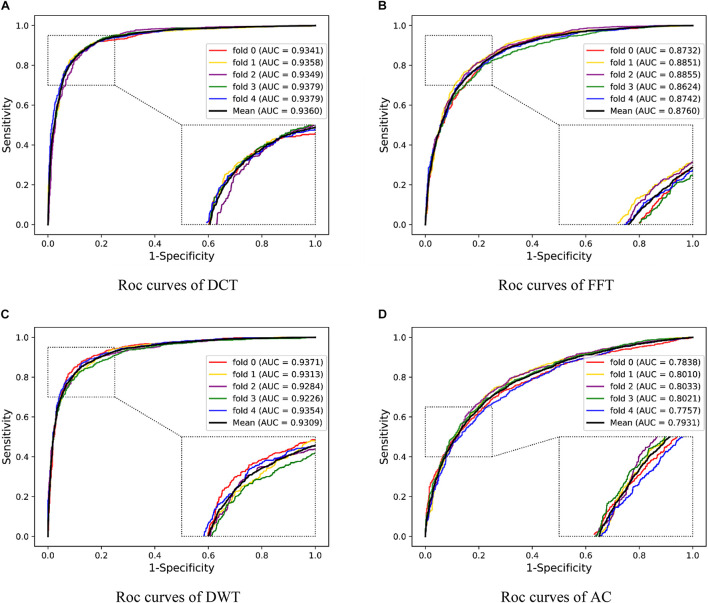
Five-fold CV results obtained by DNN classifier with different feature descriptors on the *Oryza sativa* dataset. **(A)** is the ROC curves obtained by DCT descriptors; **(B)** is the ROC curves obtained by FFT descriptors; **(C)** is the ROC curves obtained by DWT; **(D)** is the ROC curves obtained by AC.

To illustrate the effectivity of our feature extraction approach, we compared DHT with some popular correlative methods, including discrete cosine transform (DCT) (Ahmed et al., 1974), fast Fourier transform (FFT) (Nussbaumer, 1981), discrete wavelet transform (DWT) (Nanni et al., 2012), and auto-covariance (AC) (Zeng et al., 2009). As shown in Table 6 and Figure 7, on the *O. sativa* dataset, our method obtained a high prediction accuracy of 82.60%. The prediction accuracy values of other methods are 80.95, 75.31, 81.54, and 66.63%, respectively. Our method performs better than the other four methods. Especially compared with the AC-based method, our approach improved the Acc, Spec, MCC, and AUC by over 15%, and PR and Sens by over 7%, respectively. Although the Sens value of our method is not the highest, it still obtains an excellent value of 95.89%. The Acc, PR, Sens, Spec, MCC, and AUC values obtained from our model are 1.06, 0.69, 1.08, 1.05, 2.15, and 1.31% higher than the values of the DWT-based method. These comparison results further indicated the superiority of the proposed method.

**TABLE 6:** Performance comparison of the DHT with different feature extraction methods on *Oryza sativa* dataset.

Descriptors	Acc (%)	PR (%)	Sens (%)	Spec (%)	MCC (%)	AUC
DCT+DNN	80.95 ± 1.10	73.70 ± 1.41	**96.12 ± 1.15**	65.64 ± 2.40	64.99 ± 1.97	0.9360 ± 0.0017
FFT+DNN	75.31 ± 1.37	68.61 ± 1.03	93.34 ± 1.59	57.23 ± 2.90	54.26 ± 2.81	0.8760 ± 0.0096
DWT+DNN	81.54 ± 3.05	75.10 ± 3.84	94.81 ± 0.65	68.26 ± 6.61	65.50 ± 4.99	0.9309 ± 0.0052
AC+DNN	66.63 ± 4.48	62.02 ± 4.91	88.42 ± 4.77	45.02 ± 12.49	37.39 ± 5.39	0.7931 ± 0.0126
**Our method**	**82.60 ± 1.79**	**75.79 ± 2.43**	95.89 ± 0.91	**69.31 ± 3.53**	**67.65 ± 2.98**	**0.9440 ± 0.0058**

### Predictive Ability on Yeast and Human Dataset

**FIGURE 8:**
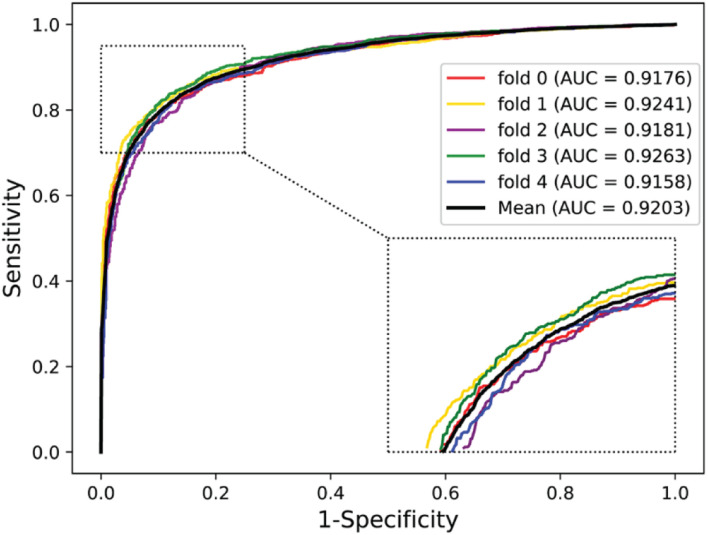
ROC curves performed by the proposed model on yeast dataset.

**FIGURE 9:**
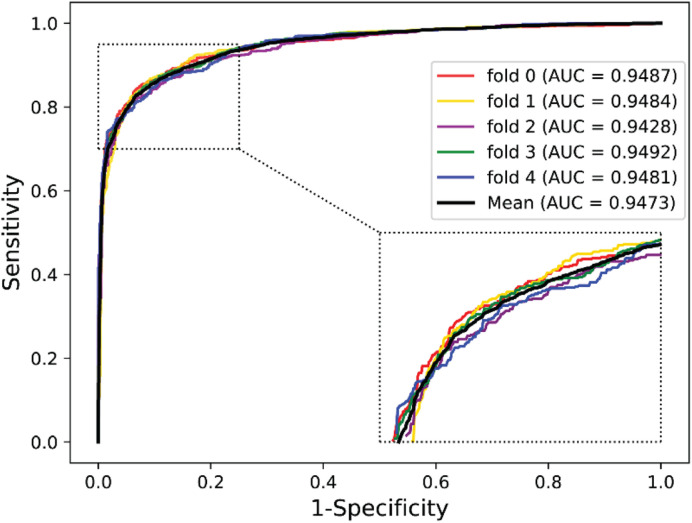
ROC curves performed by the proposed model on human dataset.

To further validate the potential of the presented method, we performed it on the yeast and human PPI dataset, which was introduced by Guo et al. (2008) and Huang et al. (2015). The predictive results of the two datasets are listed in Tables 7, 8, and the corresponding ROC curves are shown in Figures 8, 9. When performing on the yeast dataset, it achieved average Acc, PR, Sens, Spec, MCC, and AUC value of 79.54%, 73.46%, 92.63%, 66.47%, 61.27%, and 0.9203, with SDs of 1.43, 2.11, 1.18, 3.60, 2.16, and 0.46%, respectively. From Table 8, it can be observed that the proposed model yielded great results on the human dataset, an average Acc of 82.76%, PR of 75.79%, Sens of 94.18%, Spec of 72.30%, MCC of 67.72%, and AUC of 0.9473, with SDs of 1.68, 2.79, 1.64, 4.74, 2.37, and 0.26%, respectively. From these results, we can observe that the powerful DNN-based classifier combined with the DHT feature descriptor is accurate and robust for exploring cross-species predictions of PPIs.

**TABLE 7:** Five-fold CV results performed on the yeast dataset by the proposed model.

Testing set	Acc (%)	PR (%)	Sens (%)	Spec (%)	MCC (%)	AUC
1	77.20	70.38	93.33	61.31	57.60	0.9176
2	79.88	73.51	91.84	68.50	61.82	0.9241
3	79.44	73.17	93.73	64.80	61.27	0.9181
4	80.20	73.97	93.31	67.03	62.56	0.9263
5	81.00	76.27	90.95	70.70	63.09	0.9158
**Average**	**79.54 ± 1.43**	**73.46 ± 2.11**	**92.63 ± 1.18**	**66.47 ± 3.60**	**61.27 ± 2.16**	**0.9203 ± 0.0046**

**TABLE 8:** Five-fold CV results performed on the human dataset by the proposed model.

Testing set	Acc (%)	PR (%)	Sens (%)	Spec (%)	MCC (%)	AUC
1	82.41	74.69	94.07	72.28	67.18	0.9487
2	82.05	74.09	95.19	70.34	66.92	0.9484
3	83.76	78.61	92.33	75.36	68.60	0.9428
4	84.99	78.87	92.96	77.92	71.17	0.9492
5	80.59	72.70	96.36	65.59	64.75	0.9481
**Average**	**82.76 ± 1.68**	**75.79 ± 2.79**	**94.18 ± 1.64**	**72.30 ± 4.74**	**67.72 ± 2.37**	**0.9473 ± 0.0026**

## Discussion

In this article, we proposed a deep learning framework to predict PPIs in plants only using the information of amino acid sequence. This approach is based on DNN combined with DHT descriptors and PSSM. More specifically, we first used the PSSM to represent plant protein sequences, and then extracted feature vectors from these matrices by DHT. To improve the prediction accuracy and reduce the computational complexity, the SVD algorithm was adopted to reduce the feature dimensions. Lastly, these feature descriptors were sent to the DNN classifier for training and predicting. To verify the performance of the proposed approach, we performed it on *A. thaliana*, *Z. mays*, and *O. sativa* datasets. To evaluate the power of the DNN-based classifier, we compared it with the KNN and RF classifier using the same DHT descriptors. In addition, we also compared the DHT with some different feature descriptors. To further indicate the generality of our model, we also applied it to the yeast and human datasets. The experimental results indicated that our model performs significantly well in predicting PPIs in plants. In further work, we will continue to design more effective computational models for better analyzing biomolecular interactions in plants.

## Data Availability Statement

Publicly available datasets were analyzed in this study. This data can be found here: http://arabidopsis.org/; http://www.ebi.ac.uk/intact; http://www.thebiogrid.org/; http://comp-sysbio.org/ppim; http://bis.zju.edu.cn/prin/.

## Author Contributions

JP, L-PL, and Z-HY: conceptualization, methodology, software, validation, formal analysis, investigation, resources, and data curation. C-QY and Z-HR: writing – original draft preparation, writing, review, editing, visualization, and supervision. Y-JG: project administration. Z-HY: funding acquisition. All authors read and approved the final manuscript.

## Conflict of Interest

The authors declare that the research was conducted in the absence of any commercial or financial relationships that could be construed as a potential conflict of interest. The reviewer Z-AH declared past co-authorships with one of the author Z-HY and the reviewer HY-a declared past co-authorships with two of the authors Z-HY and C-QY to the handling Editor.

## Publisher’s Note

All claims expressed in this article are solely those of the authors and do not necessarily represent those of their affiliated organizations, or those of the publisher, the editors and the reviewers. Any product that may be evaluated in this article, or claim that may be made by its manufacturer, is not guaranteed or endorsed by the publisher.

## Footnotes

1
https://www.arabidopsis.org/

2
https://www.ebi.ac.uk/intact/

3
https://thebiogrid.org/

4
http://comp-sysbio.org/ppim/

5
http://systemsbiology.cau.edu.cn/agriGOv2/

6
http://bis.zju.edu.cn/prin/

7
http://blast.ncbi.nlm.nih.gov/Blast.cgi
